# A New Wavelet-Based Privatization Mechanism for Probability Distributions

**DOI:** 10.3390/s22103743

**Published:** 2022-05-14

**Authors:** Hélio M. de Oliveira, Raydonal Ospina, Víctor Leiva, Carlos Martin-Barreiro, Christophe Chesneau

**Affiliations:** 1 Department of Statistics, CASTLab, Universidade Federal de Pernambuco, Recife 50670-901, Brazil; hmo@de.ufpe.br (H.M.d.O.); raydonal@de.ufpe.br (R.O.); 2 School of Industrial Engineering, Pontificia Universidad Católica de Valparaíso, Valparaíso 2362807, Chile; 3 Faculty of Natural Sciences and Mathematics, Escuela Superior Politécnica del Litoral ESPOL, Guayaquil 090902, Ecuador; cmmartin@espol.edu.ec; 4 Faculty of Engineering, Universidad Espíritu Santo, Samborondón 0901952, Ecuador; 5 Department of Mathematics, Université de Caen Basse-Normandie, F-14032 Caen, France; christophe.chesneau@unicaen.fr

**Keywords:** artificial intelligence, data fitting, database-sensor, digital image sensor, machine learning, perturbation theory, signal-to-noise ratio, statistical modeling, wavelets

## Abstract

In this paper, we propose a new privatization mechanism based on a naive theory of a perturbation on a probability using wavelets, such as a noise perturbs the signal of a digital image sensor. Wavelets are employed to extract information from a wide range of types of data, including audio signals and images often related to sensors, as unstructured data. Specifically, the cumulative wavelet integral function is defined to build the perturbation on a probability with the help of this function. We show that an arbitrary distribution function additively perturbed is still a distribution function, which can be seen as a privatized distribution, with the privatization mechanism being a wavelet function. Thus, we offer a mathematical method for choosing a suitable probability distribution for data by starting from some guessed initial distribution. Examples of the proposed method are discussed. Computational experiments were carried out using a database-sensor and two related algorithms. Several knowledge areas can benefit from the new approach proposed in this investigation. The areas of artificial intelligence, machine learning, and deep learning constantly need techniques for data fitting, whose areas are closely related to sensors. Therefore, we believe that the proposed privatization mechanism is an important contribution to increasing the spectrum of existing techniques.

## 1. Introduction

Probability models capable of capturing the fundamental information contained in modern data, as those used for artificial intelligence [1] and big data [2], as well as models presenting unique features, have promoted derivations of novel continuous probability distributions [3,4].

Numerous and diverse approaches have been proposed over time to generate new probability or statistical distributions [5]. One of the most common approaches allows us to enhance the functionality of a base continuous cumulative distribution function (CDF). This can be achieved utilizing various transformations based on exponential, logarithmic, power, or other functions [6].

On this topic, we may refer to the so-called “families of probability distributions”, as described in [7,8]. The new probability distributions may be employed efficiently in diverse settings, as described in [9,10]. We may also refer to the work stated in [11] pointing out the importance of continuous probability distributions in the definition of various measures.

In view of the impacts of the current research on probability distributions [12], diverse applications related to the areas of artificial intelligence [1], machine learning [13], and deep learning [14] constantly require new techniques for data fitting, whose areas are closely related to sensors. Additionally, to aid in the progress of computer sciences, new approaches are welcome to expand the options of a reference probability distribution [15].

An application of probability models can be introduced by perturbing a CDF additively, similarly to how a noise perturbs the signal of a digital image sensor [16]. Surprisingly, such a strategy does not appear to have received much attention in the literature. More precisely, given a continuous CDF, one can add this function to another (the perturbation function) in such a way that the resulting function is also a continuous CDF.

To propose a manageable perturbation [17], one can employ a special, well-known function called wavelet [18,19]. Basically, such a function has a wave-like oscillation with an amplitude that starts at zero and increases or decreases before returning to zero, one or more times. Wavelets may be utilized to extract information from a wide range of data, including audio signals and images often related to sensors [20], as unstructured data. To thoroughly analyze data, wavelet sets might be used. For more information on wavelets, we refer the reader to [21,22,23]. More specifically, in [24], transients and their wavelet coefficients are modeled as mixed Laplace probability density functions (PDFs). In [25], image segmentation based on a wavelet feature descriptor and dimensionality reduction was applied to remote sensing. Thus, one could involve a wavelet function to define a valid perturbation, and then a privatized probability distribution can be obtained through theoretical and practical tools.

The main objectives of this article are to propose and derive a naive theory of an additive perturbation on a continuous probability distribution based on a wavelet approach, and to illustrate it with a sensor-related application. The use of wavelets in this probability distribution setting is original, and our findings offer up a new modeling horizon, which are examined in depth. Therefore, we offer a mathematical method for choosing a suitable probability distribution to model data by starting from some guessed-at initial probability distribution. Examples for the proposed method are also presented. For the computational experiments, we utilize a database-sensor and two related algorithms.

The rest of the article is organized as follows. Section 2 introduces the new wavelet approach. In Section 3, we discuss the choice of a perturbation for an arbitrary probability distribution. Section 4 proposes a correction for statistical moments due to the perturbation. Then, in Section 5, the generalization of the perturbation approach at further levels is presented. In Section 6, we provide an empirical application of our approach. Finally, Section 7 gives the concluding remarks.

## 2. Background and Wavelet Approach

Suppose we have a random variable *X* with a continuous CDF $F_{X}$. Let us consider an additive (functional) perturbation, denoted as ε-perturbation, so that

(1) $$F_{\mathrm{priv}}\left(x\right):=F_{X}\left(x\right)+\varepsilon \left(x\right),$$

with the CDF $F_{\mathrm{priv}}$ stated in (1) being a privatized CDF.

Note that, in the expression defined in (1), the CDF of the variable *X* has been perturbed and a new function $F_{\mathrm{priv}}$ is obtained. However, the choice of the perturbation cannot be arbitrary because it could break the requirements to deal only with a probability distribution. The following conditions must be met by the perturbation:

(C1) $\lim_{|x|\to +\infty }\varepsilon \left(x\right)=0$;
(C2) ε is derivable and satisfies $\left|d\varepsilon \left(x\right)/dx\right|\leq f_{X}\left(x\right)$, where $f_{X}$ denotes the PDF related to the CDF $F_{X}$.

The conditions (C1) and (C2) above stated guarantee that $F_{\mathrm{priv}}$ is also a CDF. This new distribution could be seen as a privatized version of the reference distribution.

To describe our new wavelet approach, some definitions need to be given. Let us begin with the mathematical definition of a wavelet.

**Definition**  **1** (Wavelet function). *A wavelet is a Lebesgue measurable function ψ(x) that is both absolutely integrable and square-integrable, such that*

(2) $$\int_{-\infty }^{+\infty }\psi \left(x\right)dx=0,$$

(3) $$\int_{-\infty }^{+\infty }\psi^{2}\left(x\right)dx=1.$$

On the one hand, from the expression established in (2), observe that the absolute value of ψ is integrable over the entire real line and its result is equal to zero (0). On the other hand, in the formula stated in (3), note that the square of ψ is also integrable over R and its result is equal to one (1). Keep in mind that, in this study, we deal with compactly supported wavelets [26], that is, the closure of the set upon which the wavelet stands non-vanishing is a compact set. Specifically, if ψ is a wavelet function, then {x:ψ(x)≠0} is a compact set, and we say ψ is a wavelet of compact support. Henceforth, we assume that support {ψ(x)}≡[a,b], which plays a crucial role in our proposal [21,27]. The next definition presents the notion of wavelet cumulative function in this setting.

**Definition**  **2** (Wavelet cumulative function). *A wavelet cumulative function is defined by*

(4) $$\Psi \left(x\right):=\int_{-\infty }^{x}\psi \left(\zeta \right)d\zeta .$$

Since only compactly supported wavelets are considered, the wavelet cumulative function given in (4) can be simplified to

(5) $$\Psi \left(x\right)=\int_{a}^{x}\psi \left(\zeta \right)d\zeta ,\;a\leq x\leq b.$$

Thus, from the expression stated in (5), the following properties can be verified:

(6) Ψ(x)=Ψ(a)=0,x≤a,

(7) Ψ(x)=Ψ(b)=1,x≥b,

(8) $$\frac{d\Psi (x)}{dx}=\psi \left(x\right).$$

Note that the properties formulated in (6)–(8) are helpful. To begin with, let us deal with the uniform distribution, denoted as U[0,1], whose CDF is given by $F_{X}\left(x\right)=x$, for 0≤x≤1, where $F_{X}\left(x\right)=0$, for x≤0, and $F_{X}\left(x\right)=1$, for x≥1. A mapping is proposed to bring the support [0, 1] of the uniform distribution to the support [a,b] of the wavelet, that is, $\left[0,1\right]\overset{\mathrm{map}}{\to }\left[a,b\right]$. Then, we propose to choose a particular perturbation ε according to

(9) $$\varepsilon \left(x\right):=\Psi_{\left[0,1\right]}\left(x\right)=\frac{1}{\left(b-a\right)}\Psi \left((b-a)x+a\right).$$

For the particular choice stated in (9), the new distribution defined in (1) has the same support as the original distribution, with no perturbation added. Furthermore, imposing the condition |ψ(t)|≤1, it follows that

(10) $$\left|\varepsilon (x)\right|\leq \frac{1}{\left(b-a\right)}\int_{a}^{(b-a)x+a}\left|\psi \left(\zeta \right)\right|d\zeta .$$

From the expression established in (10), we can guarantee that |ε(x)|≤x, for all x∈[0,1]. Therefore, the condition $F_{\mathrm{priv}}\left(x\right)\geq 0$ is assured, for all x∈[0,1]. Hence, we must determine whether $F_{\mathrm{priv}}$ is always a non-descending function or not. Thus, we examine the behavior of the corresponding PDF formulated as

(11) $$f_{\mathrm{priv}}\left(x\right)=\frac{dF_{\mathrm{priv}}\left(x\right)}{dx}=1+\frac{1}{\left(b-a\right)}\frac{d\Psi \left((b-a)x+a\right)}{dx},$$

implying

(12) $$f_{\mathrm{priv}}\left(x\right)=1+\psi \left((b-a)x+a\right),$$

where $f_{\mathrm{priv}}$ denotes the PDF related to the CDF $F_{\mathrm{priv}}$.

From the formulas given in (11) and (12), it follows that $\int_{-\infty }^{+\infty }f_{\mathrm{priv}}\left(x\right)dx=1$ and $f_{\mathrm{priv}}\left(x\right)\geq 0$, for all *x*, thereby proving that this is indeed a valid PDF to be considered. Then, this new PDF and its associated CDF might be visualized as a privatized version of the reference distribution, with the privatization mechanism being named wavelet perturbation. This is that we call “privatization analysis”.

As an example, let us first consider a compactly supported wavelet defined within [0,1] proposed in [28] and mathematically defined as

(13) $$\psi_{U}\left(x\right):=-\frac{1}{2}x\ln\left(x\right)+\frac{1}{2}\left(1-x\right)\ln\left(1-x\right).$$

**Figure 1:**
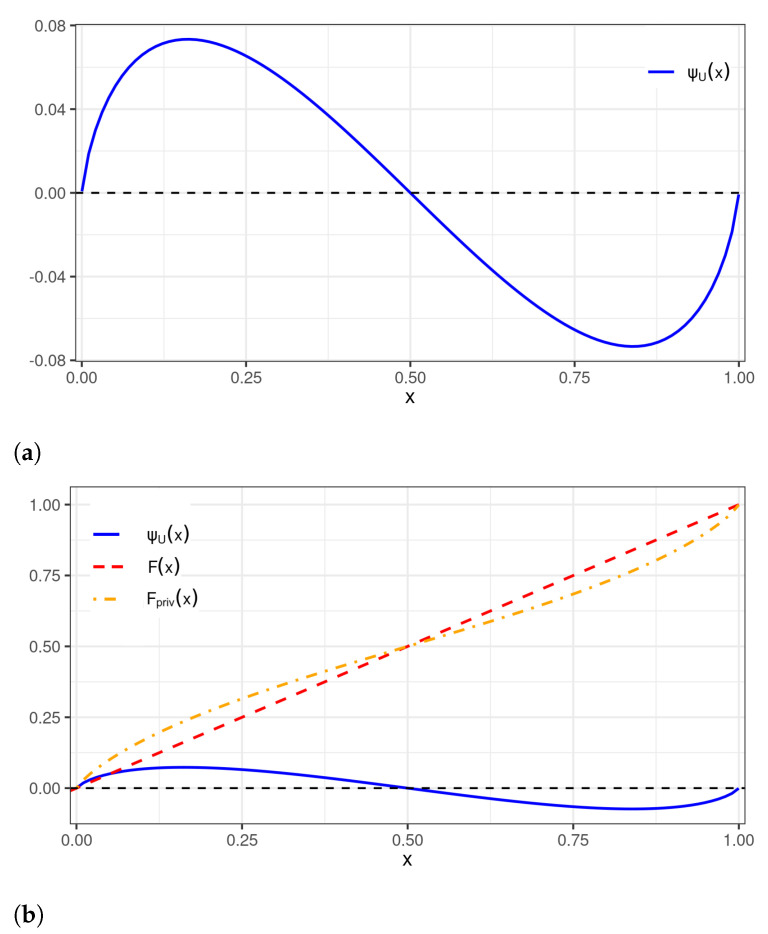
Plots of: (**a**) a wavelet perturbation to be applied to the U[0,1] distribution; and (**b**) wavelet perturbation (— blue), uniform (- - red), and perturbed uniform (- · - orange) CDFs.

Figure 1 shows the original distribution, that is, U[0, 1], and the new distribution generated by the perturbation identified in (13).

**Figure 2:**
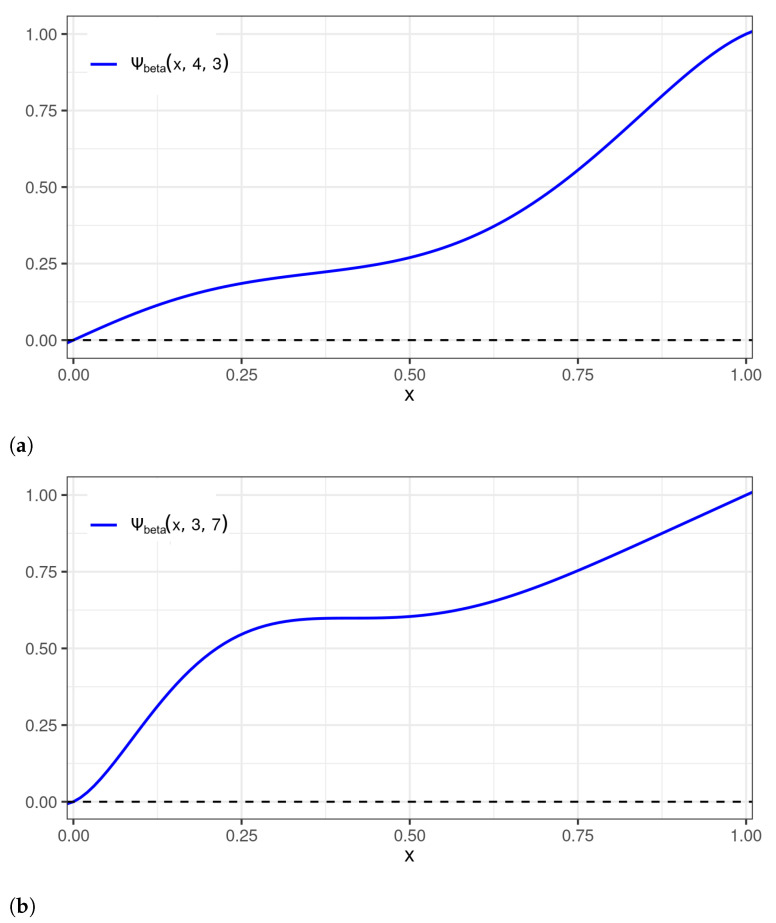
Plots of the beta wavelet perturbations: (**a**) $\psi_{\mathrm{beta}}\left(x,4,3\right)$; and (**b**) $\psi_{\mathrm{beta}}\left(x,3,7\right)$.

Another family of compactly supported wavelets with parameters that can be adjusted is the beta wavelet family [29]. One of the advantages of adopting beta wavelet perturbations consists of the easy replacement of shape (α>0) and scale (θ>0) parameters to make the perturbation $\psi_{\mathrm{beta}}\left(x,\alpha ,\theta \right)$ flexible. In other words, this wavelet family allows for a simple parametrization that drives the asymmetry of the resulting probability distribution. The plots of two beta wavelet perturbations are shown in Figure 2 as examples.

**Figure 3:**
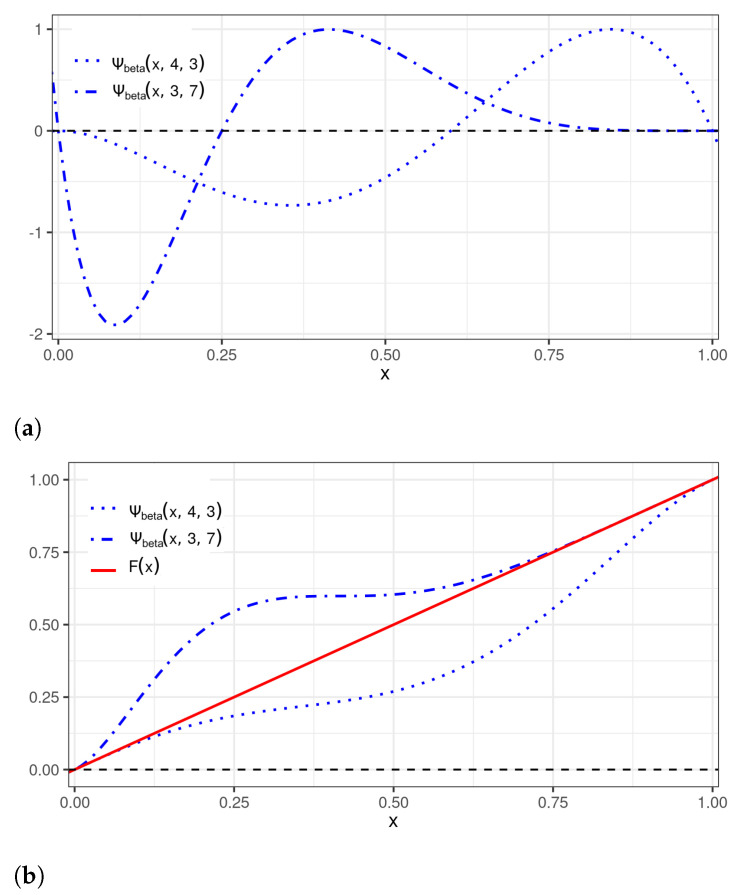
Plots of: (**a**) beta wavelet perturbations to be applied to the U[0,1] distribution; and (**b**) $\psi_{\mathrm{beta}}\left(x,4,3\right)$ perturbed uniform (⋯ blue), $\psi_{\mathrm{beta}}\left(x,3,7\right)$ perturbed uniform (- · - blue), and uniform (— red) CDFs.

**Figure 4:**
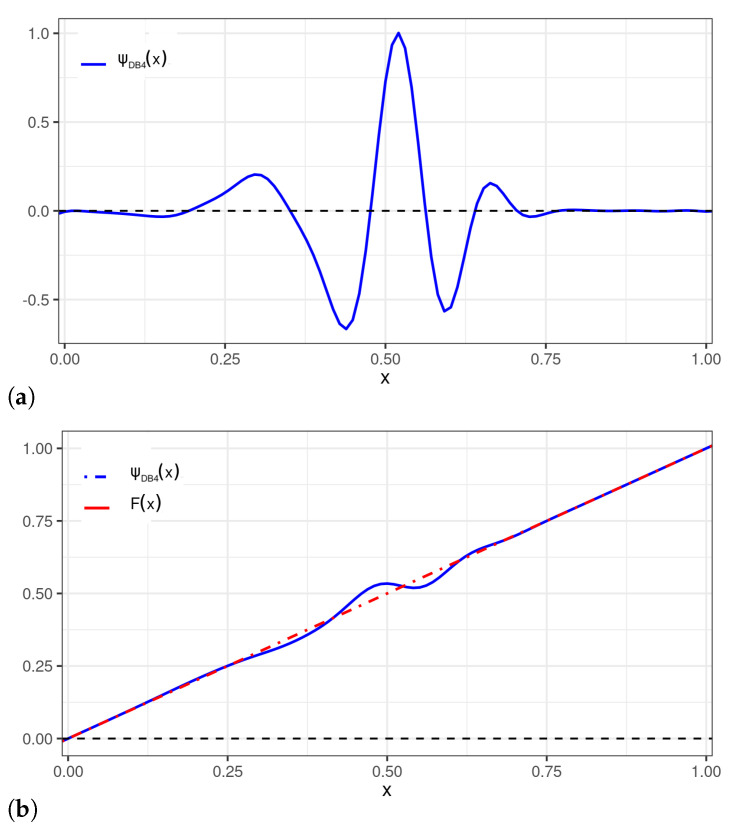
Plots of: (**a**) a DB4 wavelet perturbation to be applied to the U[0,1] distribution; and (**b**) DB4 wavelet perturbation (— blue) and uniform (- · - red) CDFs.

Figure 3 displays perturbed uniform distributions that are generated as a result of applying the perturbations of Figure 2. This approach can be employed to introduce asymmetries in a chosen probability distribution, controlled by the beta wavelet parameter. Among the compactly supported wavelets, certainly the most used are the Daubechies (DB4) wavelets [27]. Expressions close to approximately the DB4 wavelets of any order have been proposed in [30]. Using Matlab${}^{\mathrm{TM}}$ commands, these continuous approximations were employed to plot the DB4 perturbation adapted to the U[0,1] distribution, denoted by $\Psi_{\mathrm{DB}4}$, in Figure 4.

## 3. Choosing a Perturbation for an Arbitrary Probability Distribution

Now, we offer a valid perturbation for an arbitrary CDF $F_{X}$. For a given compactly supported wavelet ψ with its cumulative function (see Definition 2), consider a new chosen CDF according to

(14) $$F_{\mathrm{priv}}\left(x\right):=F_{X}\left(x\right)+\varepsilon \left(x\right),$$

with

$$\varepsilon \left(x\right):=\frac{1}{\left(b-a\right)}\frac{\Psi \left(\left(b-a\right)F_{X}\left(x\right)+a\right)}{\max_{\zeta \in [a,b]}\left|\psi \left(\zeta \right)\right|}.$$

From (11) and (14), note that $F_{\mathrm{priv}}\left(-\infty \right)=0$, $F_{\mathrm{priv}}\left(+\infty \right)=1,$ and

(15) $$f_{\mathrm{priv}}\left(x\right)=\frac{dF_{\mathrm{priv}}\left(x\right)}{dx}=f_{X}\left(x\right)+\frac{d\varepsilon (x)}{dx},$$

with dε(x)/dx stated in (15) given by

(16) $$\frac{d\varepsilon (x)}{dx}:=\frac{\psi \left(\left(b-a\right)F_{X}\left(x\right)+a\right)}{\max_{\zeta \in [a,b]}\left|\psi \left(\zeta \right)\right|}f_{X}\left(x\right).$$

Then, ε is a valid perturbation because the condition (C1) is satisfied. In addition, we have $\lim_{|x|\to +\infty }\varepsilon \left(x\right)=0$ due to $\int_{a}^{b}\psi \left(u\right)du=0$, so that the condition (C2) is also satisfied, since

(17) $$\left|\frac{\psi \left(\left(b-a\right)F_{X}\left(x\right)+a\right)}{\max_{\zeta \in [a,b]}\left|\psi \left(\zeta \right)\right|}\right|\leq 1,$$

by (16), having $\left|d\varepsilon \left(x\right)/dx\right|\leq f_{X}\left(x\right)$. Thus, any wavelet of compact support can be used to induce a different perturbation in the vicinity of the probability distribution initially assigned. From the expressions stated in (14)–(17), note that, after applying the perturbation, the resulting function is also a CDF.

In summary, given a random variable *X* with CDF $F_{X}$, a perturbation can be added, which guarantees that the modified function is still a CDF around the original CDF. This new CDF, and its associated distribution, as mentioned, are privatized versions of the reference distribution using a wavelet-based privatization mechanism.

## 4. Moments Correction Due to the Perturbation

Based on the random variable *X*, the hypothesized distribution (initial or prior distribution around which the wavelet perturbation is introduced) has its *k*-th moment defined by

(18) $$E\left(X^{k}\right):=\int_{-\infty }^{+\infty }x^{k}dF_{X}\left(x\right),$$

providing its existence in the mathematical sense. By introducing the perturbation defined in (9), the new (adjusted/privatized) *k*-th moment is stated as

(19) $$E_{\mathrm{priv}}\left(X^{k}\right):=\int_{-\infty }^{+\infty }x^{k}dF_{\mathrm{priv}}\left(x\right).$$

Consider the equation given by $dF_{\mathrm{priv}}\left(x\right)=dF_{X}\left(x\right)+\psi \left(\left(b-a\right)F_{X}\left(x\right)+a\right)dF_{X}\left(x\right).$ Then, by using the expressions given in (18) and (19), it follows that

(20) $$E_{\mathrm{priv}}\left(X^{k}\right)=E\left(X^{k}\right)+\frac{1}{\left(b-a\right)}\int_{a}^{b}{\left[F_{X}^{-1}\left(\frac{u-a}{b-a}\right)\right]}^{k}\psi \left(u\right)du.$$

The second term on the right side of (20) accounts for a moment correction due to the introduced wavelet perturbation.

Let us consider now the particular case of a perturbation in a (normalized) uniform distribution, that is, X∼U(0,1). To evaluate the moments of the new CDF $F_{\mathrm{priv}}$, under the wavelet perturbation ψ with a compact support [0,1], we have

(21) $$E_{\mathrm{priv}}\left(X^{k}\right):=E\left(X^{k}\right)+\int_{0}^{1}u^{k}\psi \left(u\right)du.$$

Note that the moment of the wavelet used to build the additive perturbation also adds to the moment of the starting distribution, because

(22) $$E_{\mathrm{priv}}\left(X^{k}\right)=E\left(X^{k}\right)+\int_{-\infty }^{+\infty }u^{k}\psi \left(u\right)du=E\left(X^{k}\right)+M_{k}.$$

If the support set is the unit interval, that is [0,1], then the formulas stated in (21) and (22) may be utilized. In the general case, if ψ has a support [a,b]≠[0,1], we can build a modified (supported-normalized) wavelet defined as

$$\psi_{\left[0,1\right]}=\frac{\psi \left((b-a)x+a\right)}{\left(b-a\right)}.$$

Hence, we have that

(23) $$E_{\mathrm{priv}}\left(X^{k}\right)=E\left(X^{k}\right)+\int_{-\infty }^{+\infty }u^{k}\psi_{\left[0,1\right]}\left(u\right)du.$$

Under the assumption that the integral term given in (23) vanishes, the moments of the new and hypothesized distributions coincide.

## 5. Generalizing the Perturbation Approach at Further Levels

In the case that a beta perturbation occurs over a U[0,1] distribution, it depends on its parameters α and θ of the perturbation wavelet. Thus, it is worth rewriting, via the equations stated in  (1)–(9), that

(24) $$\begin{array}{cc} F_{\mathrm{priv}}\left(x\right)= & \;\;\;\underset{︸}{x}\;\;\;+\underset{︸}{\Psi_{\left[0,1\right]}\left(x;\alpha ,\theta \right)}.\\ \; & \;\mathrm{approximation}\;\;\;\;\;\;\mathrm{detail} \end{array}$$

The interpretation presented in (24) of wavelet theory (approximation + detail) can be generalized into the lines of a wavelet tree with several levels. First, we present level-1 parameters (α,θ) by means of

(25) $$F_{\mathrm{level}-1}\left(x\right)=x+\Psi_{\left[0,1\right]}\left(x;\alpha ,\theta \right).$$

In Figure 3, we can see examples of this case. Second, we introduce level-2 LH parameters $\left(\alpha_{L},\theta_{L}\vdots \alpha_{H},\theta_{H}\right)$ considering

(26) $$F_{\mathrm{level}-2}\left(x\right)=\left\{\begin{array}{cc} x+\Psi_{\left[0,1\right]}\left(2x;\alpha_{L},\theta_{L}\right),\; & 0\leq x\leq 1/2;\\ x+\Psi_{\left[0,1\right]}\left(2x-1;\alpha_{H},\theta_{H}\right), & 1/2\leq x\leq 1. \end{array}\right.$$

**Figure 5:**
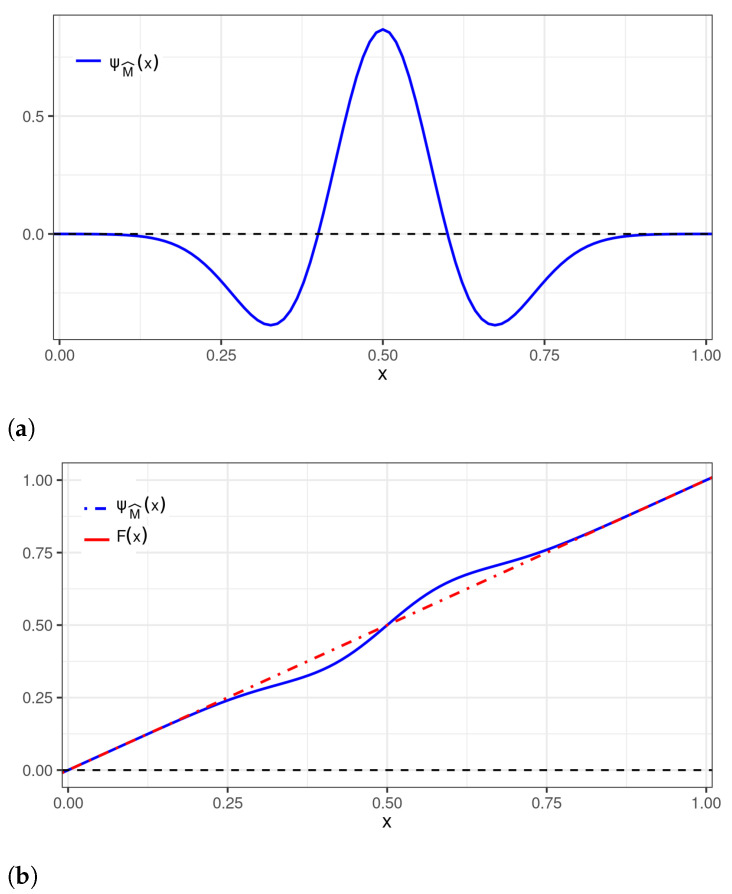
Plots of: (**a**) a Mexican-hat wavelet perturbation to be applied to the U[0,1] distribution; and (**b**) Mexican-hat wavelet perturbation (— blue) and uniform (- · - red) CDFs.

**Figure 6:**
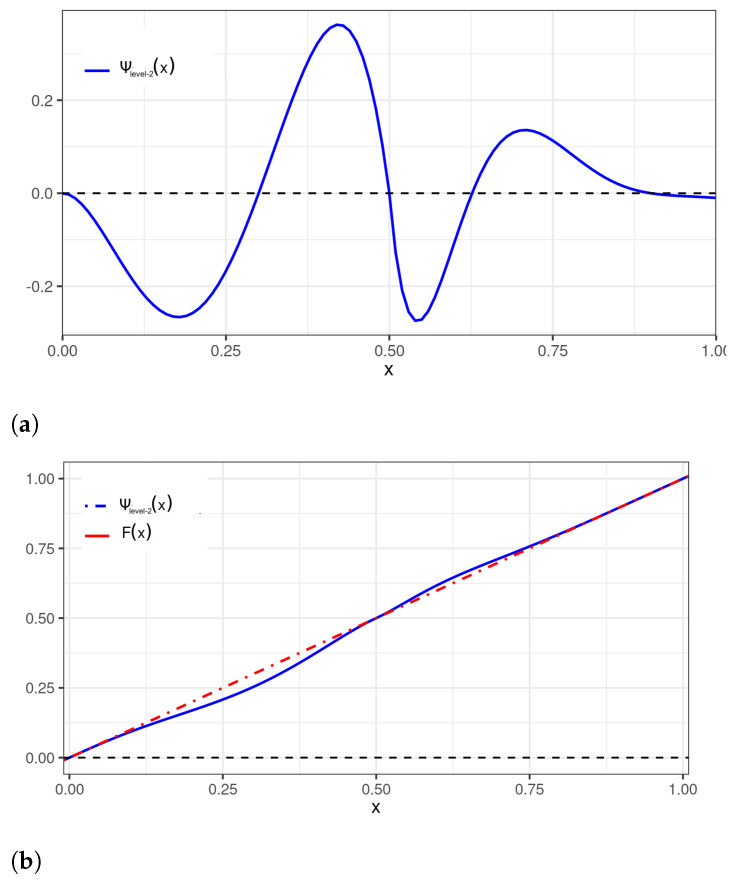
Plots of: (**a**) a level-2 beta wavelet perturbation to be applied to the U[0,1] distribution; and (**b**) level-2 beta wavelet perturbation (— blue) and uniform (- · - red) CDFs.

An example can be provided using the parameters $\alpha_{L}=4,\theta_{L}=3$, and $\alpha_{H}=3$, $\theta_{H}=7$. These parameters are similar to those employed in Figure 3. However, note that different wavelets may be selected to fit different segments of the initial distribution support. For instance, in a level-2 perturbation, the sub-level-L can use a beta wavelet, whereas the sub-level-H may employ a Mexican-hat wavelet, denoted by $\Psi_{\hat{M}}$, as in Figure 5. The parameterization $\alpha_{L}=4,\theta_{L}=3$, and $\alpha_{H}=3,\theta_{H}=7$ is used in Figure 6, with the corresponding perturbation denoted by $\Psi_{\mathrm{level}-2}$.

Next, we present level-4 LL LH HL HH parameters, $(\alpha_{\mathrm{LL}},\theta_{\mathrm{LL}}:\alpha_{\mathrm{LH}},\theta_{\mathrm{LH}}\vdots \alpha_{\mathrm{HL}},\theta_{\mathrm{HL}}:\alpha_{\mathrm{HH}},$$\theta_{\mathrm{HH}})$ namely, stated as

(27) $$F_{\mathrm{level}-4}\left(x\right)=\left\{\begin{array}{cc} x+\Psi_{\left[0,1\right]}\left(4x;\alpha_{\mathrm{LL}},\theta_{\mathrm{LL}}\right), & 0\leq x\leq 1/4;\\ x+\Psi_{\left[0,1\right]}\left(4x-1;\alpha_{\mathrm{LH}},\theta_{\mathrm{HL}}\right), & 1/4\leq x\leq 1/2;\\ x+\Psi_{\left[0,1\right]}\left(4x-2;\alpha_{\mathrm{HL}},\theta_{\mathrm{HL}}\right), & 1/2\leq x\leq 3/4;\\ x+\Psi_{\left[0,1\right]}\left(4x-3;\alpha_{\mathrm{HH}},\theta_{\mathrm{HH}}\right), & 3/4\leq x\leq 1. \end{array}\right.$$

An example of this level-4 approach is illustrated utilizing the values given by

$$\left(\alpha_{\mathrm{LL}},\theta_{\mathrm{LL}}:\alpha_{\mathrm{LH}},\theta_{\mathrm{LH}}\vdots \alpha_{\mathrm{HL}},\theta_{\mathrm{HL}}:\alpha_{\mathrm{HH}},\theta_{\mathrm{HH}}\right)=\left(4,3:\;3,7\;\vdots \;5,3:\;2,7\right).$$

An interpretation for this approach is considering a distinct perturbation in each quartile of the distribution such as:

- First quartile driven by $\left(\alpha_{\mathrm{LL}},\theta_{\mathrm{LL}}\right)=\left(4,3\right)$.
- Second quartile driven by $\left(\alpha_{\mathrm{LH}},\theta_{\mathrm{LH}}\right)=\left(3,7\right)$.
- Third quartile driven by $\left(\alpha_{\mathrm{HL}},\theta_{\mathrm{HL}}\right)=\left(5,3\right)$.
- Fourth quartile driven by $\left(\alpha_{\mathrm{HH}},\theta_{\mathrm{HH}}\right)=\left(2,7\right)$.

In short, the privatization mechanism allows us to perturb a probability distribution employing levels (applying a partition on the compact support), which may be very attractive when fitting data. We can use the expression stated in (25) when implementing one level, in (26) when implementing two levels, and in (27) when implementing four levels.

## 6. Empirical Application

Next, we apply our privatization approach to a real-world problem. An e-commerce company sells products on the Internet and wants to analyze the possibility of adding more servers or changing its most important server. By collecting daily data, we find many days in which the best server has almost all its hardware resources consumed 70% of the time. Looking at the empirical PDF and CDF, we see that a triangular distribution, with support on the set [0, 1] and mode equal to 0.7, might represent the data well. However, when performing goodness-of-fit tests, the results tell us that a triangular distribution is not the best option. However, a “quasi-triangular” distribution could be an appropriate probability model for the random variable *X* that measures the daily proportion of times with full resource consumption of the best server. Among the known techniques to fit data, the privatization mechanism that we propose in this work is an excellent option to slightly perturb the triangular distribution and describe the data well. For the computational experiments, we utilize a database-sensor and two related algorithms.

Let *X* be a continuous variable, which is triangularly distributed, with support on the interval [0, 1], and whose mode is *m*, for 0<m<1. The PDF and CDF of *X* are, respectively, given by

$$f_{X}\left(x\right)=\left\{\begin{array}{cc} \frac{2x}{m}, & 0\leq x\leq m;\\ \frac{2(1-x)}{1-m}, & m<x\leq 1; \end{array}\right.$$

and

$$F_{X}\left(x\right)=\left\{\begin{array}{cc} \frac{x^{2}}{m}, & 0\leq x\leq m;\\ 1-\frac{{\left(1-x\right)}^{2}}{1-m}, & m<x\leq 1. \end{array}\right.$$

**Figure 7:**
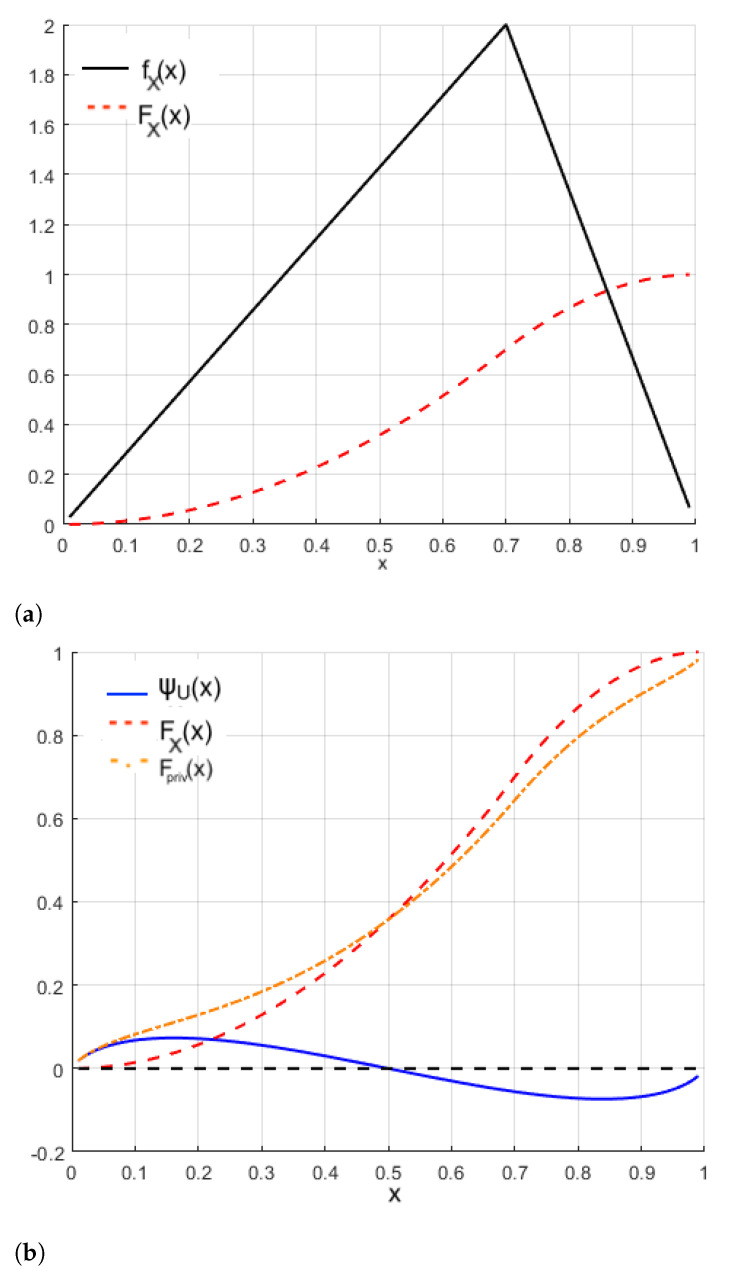
Plots of: (**a**) PDF and CDF of the triangular distribution; and (**b**) wavelet perturbation (— blue), triangular (- - red), and perturbed triangular (- · - orange) CDFs.

Now, we use the wavelet function defined in (13). Figure 7 shows the graphical plot of the CDF corresponding to *X* (original triangular distribution) and also the graphical plot of the privatized version that corresponds to the random variable $X_{\mathrm{priv}}$ (perturbed triangular distribution). We consider the value m=0.7 in the calculations carried out. Note that, in the perturbed triangular distribution, the CDF values are greater than when compared to the original triangular distribution, for values of *X* less than 0.5, while for values of *X* greater than 0.5, the opposite occurs. This behavior is due to the wavelet function employed in such an empirical application. In practice, this method is flexible allowing us to choose the most convenient wavelet to fit the data.

For the computational experiments that were carried out, a database-sensor was used. Algorithm 1 shows the steps to perturb a probability distribution with compact support. If a perturbation by levels is required, we propose Algorithm 2 as a generalization of Section 5, where the number *k* of levels is left to the consideration of the data analyst.
**Algorithm 1** Approach to perturb a probability distribution with a database-sensor.1:Consider a random variable *X* with compact support [a,b].2:Select a wavelet with compact support [a,b] to perturb the distribution of the previous step, with the computations being performed by a first process denoted by A that sends the generated data to a database.3:State a sensor in the database that detects the entry of new data, so that, using a trigger, the sensor responds sending a copy of the stored data to a second process denoted by B.4:Establish that process B receives the perturbed data and is responsible for building the CDF of the resulting distribution.5:Confirm that process B generates the corresponding plots showing, between *a* and *b*, the original distribution, wavelet used, and perturbed distribution.

**Algorithm 2** Approach to perturb a probability distribution by levels.
1:Select a probability distribution with compact support [a,b].2:Apply a partition of *k* subintervals over the interval [a,b] (not necessarily equispaced).3:Use Algorithm 1 on the interval $\left[a_{i},b_{i}\right]$, for each *i* from 1 to *k*.4:Perform computations to unify the results on the interval [a,b] of the previous step.5:Generate unified plots on the interval [a,b] for the original distribution, wavelet used, and perturbed distribution.


## 7. Concluding Remarks

This paper has presented a new method for building an additive wavelet-based perturbation, as a privacy mechanism, to modify a given continuous probability distribution. Then, the initial guess could be perturbed as some sort of “prospecting within the ensemble of possible probability distributions around the starting distribution”.

The method we have proposed in this investigation is flexible with respect to the perturbation function that may be employed to fit the data, since different wavelets are available. A procedure was also offered to employ four different perturbations, one in each quartile of the original distribution, which can be quite attractive when fitting data. Examples of the proposed method were discussed. Computational experiments were carried out using a database-sensor and two related algorithms. Several knowledge areas can benefit from using the new method proposed in this study.

Stochastic programming, simulation studies, and multivariate analysis [31,32,33,34], among other areas of knowledge, may also benefit from the utilization of the new approach proposed in this investigation. The Internet of things, robotics, monitoring stations, telemetry, and the use of sensors are also important fields for data reading and fitting. Concrete applications via this new approach may now emerge, with an efficient configuration for the involved functions. Another benefit of this technique is its ease of implementation in any programming language. Software developers must be the first to get involved to make this technique available to data analysts. The areas of artificial intelligence, machine learning, and deep learning [35] constantly require new techniques for data fitting, whose areas are closely related to sensors. Accordingly, we think that the proposed privatization mechanism is an important contribution to increasing the spectrum of existing techniques. An avenue of future work to be considered is to provide a method that allows us to determine the most appropriate wavelet during data fitting.

## Data Availability

Not applicable.
